# Cognitive Impairment and Affective Disorders in Patients With Obstructive Sleep Apnea Syndrome

**DOI:** 10.3389/fpsyt.2018.00357

**Published:** 2018-08-07

**Authors:** Radoslav G. Bilyukov, Martin S. Nikolov, Ventsislava P. Pencheva, Daniela S. Petrova, Ognian B. Georgiev, Tsanko L. Mondeshki, Vihra K. Milanova

**Affiliations:** ^1^Department of Propaedeutics of Internal Diseases, Medical University of Sofia, Sofia, Bulgaria; ^2^Clinical Psychologist, Sofia University, Sofia, Bulgaria; ^3^Department of Psychiatry, Medical University of Sofia, Sofia, Bulgaria

**Keywords:** cognitive impairment, affect, sleep apnea, daytime sleepiness, apnea/hypopnea index

## Abstract

Sleep-related breathing disorders could be accompanied by or caused by a variety of medical conditions. They are considered to be a significant medical and social problem. Together with excessive daytime sleepiness, patients with obstructive sleep apnea experience neuropsychological symptoms such as anxiety, attention deficits, cognitive impairment, depressive symptoms and other psychological disturbances leading to social adjustment difficulties. Patients diagnosed with obstructive sleep apnea demonstrate a decline in a wide spectrum of cognitive abilities, including memory, attention, psychomotor speed, executive, verbal and visual-spatial skills. The aim of this study is to investigate the cognitive functioning and affective disorders among patients with obstructive sleep apnea syndrome and to examine the frequency and severity of cases in comparison with a control group consisting of healthy volunteers. Our research has shown that there is a relation between sleep apnea and cognitive impairments and affective changes. This relation can be explained by the direct effect of the syndrome on the patient, where the main connecting factor is the severity and the distribution of excessive daytime sleepiness. Along with treatment of the somatic medical condition, it is extremely important that the patient's mental state is treated as well.

**Trial Registration:** 57/2013, Medical University - Sofia, Bulgaria.

## Introduction

Sleep-related breathing disorders could be accompanied by or caused by a variety of medical conditions. These disorders are considered to be a significant medical and social problem. The most common breathing disorder subtype during sleep is obstructive sleep apnea syndrome (OSA), which is characterized as a chronic condition; due to the disruption of sleep architecture, OSA is the main cause of excessive daytime sleepiness, increased cardiovascular risk, metabolic disorders, and cognitive and affective changes.

Obstructive sleep apnea syndrome consists of a range of symptoms, caused by intermittent, repetitive episodes of complete cessation or reduction of the upper airway airflow during sleep. Obstructive sleep apnea is defined as lack of oronasal airflow for a period of at least 10 s and at least 4% desaturation, while chest and abdominal respiratory efforts still continue. In comparison to obstructive sleep apnea, obstructive hypopnea is characterized by a 50% reduction of the normal airflow with the same duration and desaturation criteria as OSA, along with sleep fragmentation. Both disorders are commonly accompanied by arousals, which disturb the sleep architecture (1).

Snoring and excessive daytime sleepiness (EDS) are significant symptoms that could lead clinicians to presume the presence of OSA (2, 3). Overnight polysomnography (PSG) is the only objective measurement method used as a gold standard in the diagnosis of OSA. PSG is performed by qualified professionals within specialized medical centers. PSG allows data recording and differentiation of the breathing disorders, providing tools to compare the data with the records of the sleep stages and arousals. The method gives a vast amount of information about the main vital signs and their degree of disturbance. It is used for the diagnosis of many types of sleep disorders—narcolepsy, periodic limb movements, parasomnias, insomnias, sleep-related apnea.

An important parameter in the investigation process of OSA is the Apnea-Hypopnea Index (AHI), which represents the number of apneas and hypopneas per hour of sleep. According to the AHI-values, OSA could be categorized into three degrees of severity: mild, moderate and severe. AHI decreases and approaches normative values (below five), following an appropriate treatment of OSA (1, 4).

In addition to excessive sleepiness, patients with OSA also experience neuropsychological symptoms such as anxiety, attention deficits, cognitive impairment, depressive symptoms and other psychological disturbances leading to social adjustment difficulties. Patients diagnosed with OSA demonstrate a decline in a wide spectrum of cognitive abilities including memory, attention, psychomotor speed, executive, verbal and visual-spatial skills (5).

The aim of this study is to investigate the cognitive functioning and affective disorders among patients with obstructive sleep apnea syndrome and to examine their frequency and severity in comparison with the scores of a control group, consisting of healthy volunteers.

## Materials and methods

### Materials

The current study includes 76 participants—adults of legal age, divided in two groups as follows: patients with OSA and a control group. The patients' group consists of 53 people, while the number of participants in the control group is 19. All participants in the research were volunteered and signed a written informed consent, approved by the local ethics committee. The informed consent provided information about the procedures and possible risks and benefits of the study. It also guaranteed their freedom to end participation at any time.

Patients with obstructive sleep apnea syndrome were diagnosed by night time polysomnography. Patients with inflammatory and malignant lung diseases, pneumothorax, acute or chronic respiratory failure, as well as patients who had formerly undergone continuous positive airway pressure (CPAP) treatment were not included in the research group. Additional exclusion criteria for participation are severe cardiovascular diseases, including a severe-degree arterial hypertension, disorders of the cardiac rhythm and conductivity, acute myocardial infarction, unstable angina pectoris, severe heart failure, patients who have suffered a cerebrovascular accident, inflammatory diseases, chronic viral hepatitis and liver cirrhosis, liver insufficiency, or chronic kidney insufficiency. Immunocompromised patients were excluded from the current research as well (patients with neoplasms, after organ transplantation, AIDS, hematological disorders, inherited immunodeficiency disorders, systemic connective tissue disorders). Patients with alcohol or drug addiction and patients with severe mental disorders (schizophrenia, bipolar affective disorder with severe symptoms, severe depressive episode with or without psychotic symptoms, mania episodes, recurrent depression, as well as pregnant women and patients with severe uncontrolled endocrine pathology) were also excluded. Patients diagnosed with the aforementioned medical conditions during the course of the research were excluded.

The participants in the control group are clinically healthy subjects with clear anamnestic data on sleep-related breathing disorders and normal electrocardiogram and overnight pulse oximetry results. The rest of the exclusion criteria are identical for both groups.

Additional exclusion criteria for both groups are working on shifts and the presence of medical conditions, related to disorders of the circadian sleep-wake rhythm.

The participants in the test groups are patients of the Laboratory for Sleep Apnea and of the Clinic for Propedeutics of Internal Diseases of the University Hospital “Alexandrovska,” involved in the research in 2014–2016.

The control group is composed of people from the common population, who were recruited during the same period.

During the tests for admitting the subjects to the research, a total of four people was excluded: three from the group diagnosed with OSA and one from the control group. The reasons for that decision were neurological organics, a genetic disorder, a psychiatric disorder (bipolar affective disorder) and circadian rhythm disorders. The excluded people have not been included in the total number of participants.

### Methods

#### Anamnesis and general health status

Anamnesis and general health status (for all the participants), including anthropometric data—age, sex, height, weight, body mass index (BMI).

#### Instrumental

- Electrocardiogram—standard, 12-channel (to exclude substantial cardiovascular pathology).
- Polysomnography—a 64-channel polysomnograph Compumedics was used. The polysomnogram incudes channels, which record brain activity—electroencephalography (EEG), eye-movement (EOG), muscle tone (EMG) cardiac rhythm (electrocardiogram—ECG), oronasal airflow, pulse oximetry. The data from each channel are recorded and stored. During sleep, the computer provides data-output for all channels used.

EEG is used to register the neuronal activity of the brain during sleep and to classify the different sleep stages—phase 1, 2, 3, 4 (non-REM), the R-stage (REM-sleep), and the awake-stage. The EEG electrodes are placed according to all guidelines and acknowledged standards.

The EOG is used for registration of REM-sleep, as well as the very beginning of the sleep process.

The EMG helps to register the sleep onset. The latter is defined by general relaxation and significant decrease in the muscle tone of the body. During REM-sleep deeper muscle tone relaxation is observed.

The airflow is registered by transducers for measuring pressure or with the help of thermistors, placed near the nostrils, thus enabling the measurement of the frequency of apneas during sleep.

The pulse oximetry registers the fluctuations in the oxygen saturation, which occur in patients with sleep apnea.

Sleep apnea is diagnosed with the help of a polysomnography record under the premise that more than five episodes of obstructive or central apneas/hypopneas per hour (AHI—Apnea-Hypopnea Index) have been recorded. Based on overnight polysomnography and the Apnea-Hypopnea Index and according to the international guidelines, the disease is categorized in three degrees of severity—mild (AHI = 5–14.9), moderate (AHI = 15–29.9) and severe (AHI > 30).

- Night pulse oximetry—performed with a Nonin Medair LS1-9R—a percutaneous pulse oximetry and capnography device used for measuring and recording the oxygen saturation and partial pressure of carbon dioxide in the exhaled gases. This is a screening method to help exclude breathing disorders during sleep (respectively, desaturation episodes) and is applied for the participants in the control group.

#### Psychological tests

##### The epworth sleepiness scale

The Epworth Sleepiness Scale (ESS) measures the overall daytime sleepiness or the average sleep propensity in daily life. ESS has become the world standard as a method to calculate daytime sleepiness. The questionnaire consists of eight statements. Each statement is assigned a number between 0 and 3. The statements describe eight different daily situations in which one may quite possibly doze off or fall asleep. The test takes approximately 2–3 min. The maximum total score is 24, where a score of seven or more points is an indication for evident daytime sleepiness. A score of nine points and more is related to clinically significant levels of daytime sleepiness. The current research uses the standardized Bulgarian version of the test (6).

##### Word memory test

The currently applied memory test is the Ten Words Memory Test of Alexander Luria. The method allows assessment of general cognitive functioning, including sustained attention and experienced tiredness during the research process. Three memory components are tested: fixation, reproduction and retention. This method is divided into three stages. The first concerns memorizing capacity, or fixation; it consists of a list of 10 words, read aloud in five consecutive trials. At this stage the participant is asked to memorize and recall the words from the list. The second stage investigates the reproduction, and the third examines retention. Approximately 1 h later the subject is asked to recollect and repeat the list (reproduction). At the last stage, the researcher reads the test material one more time and the participant repeats its content immediately (retention). Two sets of 10 words have been used as stimulus materials. Set 1: ball, bee, fire, coat, pear, plate, bird, beer, dog, river. Set 2: water, sun, pickaxe, hay, suitcase, string, rabbit, hat, steam, cat. Scoring is separately calculated for every single stage. Scores under 84% for any of the stages indicate memory disturbances (7).

##### Mini-mental state examination (MMSE)

The MMSE is considered to be a reliable screening method used to differentiate the cognitively impaired individuals from the healthy ones. The MMSE offers a systematic and profound assessment of the psychic status. The questionnaire includes 11 items that evaluate five cognitive domains: orientation, registration, attention and calculation, recall and speech. The scale is effective for screening hospitalized adults with cognitive disturbances. The test administration requires 5–10 min. Each question is scored specifically. The maximum score for the MMSE is 30. A result of 23 points or less is indicative for cognitive impairment. Since it was published in 1975, the MMSE has been renowned and widely used in both clinical practice and clinical research. The standardized Bulgarian version from 29.07.2008 was used in the current research (8).

##### Trail making test

The trail making test (TMT) requires quick recognition of the symbolic meaning of numbers and letters, and the task is to draw lines as quickly as possible to connect the elements in sequence, with different pages and finding the next symbol, flexibility to integrate alphabetic and numeric sets of symbols, and performing these tasks under the pressure of time limitation. TMT gives information about the attention, psychomotor speed, executive functions and general cognitive functioning. The test includes two parts—part A and B. The version for adults, wherein both parts consist of 25 circles containing a symbol, was used in the current research. The task in part A is to connect the 25 circles that contain the numbers from 1 to 25 in sequence, starting from 1 and connecting the circles in the correct order. In part B the subject must connect in a sequential order the 25 circles, containing numeric and alphabetic symbols. The two parts were scored separately, and the duration of each part was recorded. The errors were also noted, but they do not directly affect the score of the subject. For part A, a duration of 39 s or more is considered to indicate a mild-to-moderate cognitive impairment. For part B, a duration of more than 85 s is indicative of cognitive impairment. For the purposes of the current research, the standardized Bulgarian version from The Neuropsychology Center has been used. The version used is from 2008 (8, 9).

##### Zung self-rating depression scale

The self-rating depression scale is a short, self-administered questionnaire used to assess the degree of depressive symptoms (the degrees can be mild, moderate or severe). The questionnaire contains 20 statements, which assess affective and somatic symptoms associated with depression. Each statement is scored on a scale from 1 to 4 based on the answers “a little of the time or never,” “some of the time,” “good part of the time,” “most of the time or all of the time.”

The scores range from 20 to 80. Scores of over 44 points indicate the presence of depressive symptoms. In this research, a standardized Bulgarian version of the scale is used (10).

##### Neurotic-depressive test (NDT) of T. Tashev

NDT is a self-rating questionnaire used to determine the level of anxious and depressive states (the degree can be mild, moderate or severe). The questionnaire includes 69 statements that assess affective and somatic symptoms associated with anxiety and depression. A positive or negative reply is given to each statement. Only positive replies are scored. The results of the test range from 0 to 69. Results over 17 indicate the presence of anxiety-depression symptoms. The original version of the questionnaire was used in the current research (11).

#### Statistical methods

The statistical analysis of the results was performed with the IBM SPSS Statistics v. 19 for the Windows 7 operating system. A statistical significance level of *p* ≤ 0.05 indicates that the null hypothesis should be rejected. The following methods were used: analysis of variance of quantitative variables (mean value and standard deviation, standard error), correlation analysis, regression analysis, and graphic output. To determine a correlation between two qualitative variables, Fisher's exact test was used.

## Results

### Characteristics of the anthropometric data

There are 53 patients in the experimental group, including 8 women (15.09%) and 45 men (84.91%). The average age of the group is 49.42 ± 11.78 years, and the average body mass index is 37.39 ± 4.83.

The control group includes 19 healthy individuals, including 11 men (57.89%) and 8 women (42.11%). The mean age in this group is 45.53 ± 13.07, and the average BMI is 33.35 ± 4.18.

All controls and patients are of similar age and body mass index. The fact that men are more frequently prone to OSA explains the prevalence of male individuals among the patients' group.

### Statistical properties of sleep architecture

The analysis of sleep architecture includes an account of average sleep duration and average duration of the single sleep stages (non-REM stages 1, 2, 3, 4, and REM stage). Arousal index and sleep efficiency are also taken into account. Analysis of the average pre-sleep period (sleep latency) and pre-REM period is included.

The average sleep efficiency among the patients is 81.6% (±16.13). The lowest and highest efficiencies are 35 and 98.6%, respectively. The average arousal index amounts to 37.26 (±25.17).

Average sleep latency is 3.84 min., and the pre-REM duration is at an average of 109.95 min (±101.99).

The average length of the non-REM stages among patients with OSA are 10.2% ±8.94 for stage 1, 44.08% ±16.77 for stage 2, 30.91% ±16.02 for stage 3 and 11.49% ±13.09 for stage 4. The average length of the REM-stage is 3.31%.

Sleep architecture among patients with OSA is characterized by fragmentation, arousals, prevalence of the superficial sleep stages (mainly stage 2 of non-REM sleep), reduced REM sleep and reduced deep slow-wave sleep (stage 4 of non-REM). The polysomnographic properties presented herein represent a constellation of variables that allow the quality of sleep to be assessed.

### Statistical characteristics of the breathing disorder

The analysis of the breathing disorder is based on the Apnea/Hypopnea Index; the Obstructive/Central/Mixed Apnea and Hypopnea Index; average and minimum oxygen saturation; computing the relative amount of time spent sleeping while the saturation of the patient is under 90%.

The average value of Apnea/Hypopnea Index among patients with sleep related breathing disorders is 58.39/h. Most of the patients demonstrate the obstructive type of the apnea or hypopnea disorder. The apnea index is 33.683 ± 28.677 and hypopnea index is 24.596 ± 15.155. Mixed Apnea Index and Central Apnea Index express the number of breathing disturbances per hour; their current average values are 2.02 and 0.33, respectively.

Using the Apnea/Hypopnea Index as an integral index for respiratory events during sleep, the patients can be divided into three groups according to the degree of the disorder: mild (AHI 5–14.9), moderate (15–29.9) and severe (above 30). The cohort of OSA patients consists of 3 cases (5.7%) of mild degree; 7 cases (13.2%) of moderate degree and 43 cases (81.1%) of severe degree.

The average level of desaturation monitored during apnea/hypopnea events is 10.2660% (±4.72), with a minimum level of desaturation of 3% and maximum 20%. The time of sleep with saturation under 90% for the whole group is at an average of 50% from the sleep, whereas the minimum duration is 5.1% and the maximum is 99.9%.

The lowest recorded saturation is 45%, whereas the average for the group is 62.9% ±16.838. The time of sleep with saturation under 90% is 52.320 ± 29.830 min.

### Statistical properties of the cognitive status of the participants

#### Daily sleepiness

After the ESS examination of the group of OSA patients, the results show that 18 of the patients have a severe form of daytime sleepiness (34%) 22 have a mild or moderate form (41%), and the rest of the examined (25% of the group) are healthy or in the norm, respectively 8 and 5 cases. The ESS mean for the group is 12.75 (±5.4). In comparison to the OSA patient group, the control group has statistically lower results for ESS, *p* < 0.001.

Based on the degree of OSA severity in the OSA patient group, a trend is apparent—the average results of the examined subjects with mild (9 ± 1) and moderate (9.67 ± 5.27) degree is lower than the average of those with severe OSA (13.7 ± 5.73), but the trend is statistically insignificant (*p* = 0.14). Among those examined with AHI > 30, 24 people show symptoms of daytime sleepiness−13 of them (43%) with severe daytime sleepiness, 11 (37%) with a mild or moderate degree. A total of six people from the group (20%) are healthy or within normative values. Among the patients with moderate and mild OSA, 1 (11%) demonstrates severe daytime sleepiness, 3 demonstrate mild or moderate daytime sleepiness (33%), and 5 are healthy or within the normative values (56%).

**Figure 1:**
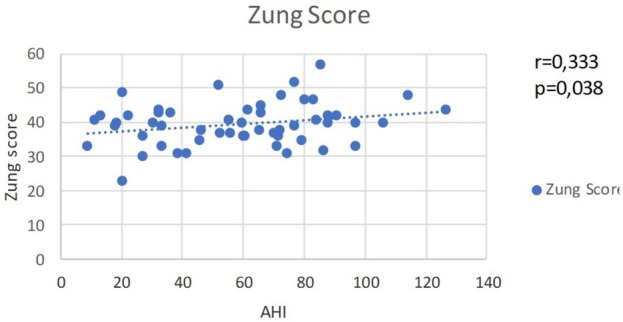
Scores of ESS and AHI by cases that show direct moderate correlation between OSA severity and daytime sleepiness.

Based on the correlative analysis of the test results for ESS and AHI, the following correlation is present: a moderate positive correlation (*r* = 0.333, *p* < 0.038), which affirms the association between OSA and daytime sleepiness symptoms and confirms ESS as a standard for diagnosing OSA (Figure 1).

For the OSA patient group, memorizing ability (fixation), measured with the 10 Words Memory Test, showed mean scores of 76.17 (±10.42). According to the results, 7 people (16.7%) are healthy, 27 (64.4%) are with a mild impairment, 6 (14.3%) are with moderate impairment and 2 (4.8%) are with severe impairment of memory functions. There is no statistically significant relation between the means of AHI scores and the results from the memory test: severe OSA −75.54 (±10.33), moderate OSA −74.67 (±15.47), mild OSA −79.33 (±9.45) (*p* = 0.831).

Among the participants with severe OSA as per the AHI scores, 1 (4%) demonstrates severe impairment, 6 (21%) demonstrate a moderate impairment, 17 (61%) demonstrate a mild impairment of the memorizing ability, and 4 (14%) are healthy.

The distribution among patients with mild-to-moderate AHI scores is as follows: 1 patient (11%) with severe impairment, 6 (67%) with mild impairment and 2 (22%) healthy. The mean of the control group is 83.23 (±9.85), which is significantly higher than the score of the OSA group and indicates a better memorizing ability (*p* < 0.035). The relation between the control group and the group of patients with high AHI scores is identical (*p* < 0.03).

The mean value of the results of the OSA group from the second part of the 10 Words Memory Test concerning the reproductive memory ability (reproduction) is 79.95 (±16.08). The results of the group show that 4 (9.8%) demonstrate a severe impairment of reproductive memory ability, 6 (14.6%) demonstrate a moderate impairment, 11 (26.8%) a mild impairment, and 20 (48.8%) are healthy. In relation to the severity of OSA, the mean values of the groups are 96.67 (±5.77) for the patients with a mild degree, 74.2 (±21.78) for those with a moderate one and 80.57 (±15) for the patients with a severe degree. A statistically significant correlation between the mean values of the three groups is not observed.

Among the patients with mild-to-moderate degree there is one patient in every category of severe, moderate and mild impairment of the reproductive memory ability [a total of 3 (33%) of the group] and 5 who are healthy (57%). In the group of patients with a severe degree of OSA 2 (7%) demonstrate a severe memory reproduction impairment, 4 (14%) show a moderate impairment, 8 with mild impairment (29%) and 14 (50%) are healthy. In comparison to the whole group of OSA patients, the controls demonstrate a mean value of 88.92 (±14.06), which indicates a tendency for better reproductive memory abilities, but the tendency is statistically insignificant (*p* = 0.077).

When examining the memory retention function all patients (both OSA group and control group) show maximum results with the exception of one case, but this case is within the normative values. There is no statistically significant correlation between the mean values of the examined variable of both groups (*p* = 0.574).

The mean value of the scores of the OSA group on the MMSE examination is 26.91 (±2.58). The results show that 8 persons from the group (18%) have mild cognitive impairments, 8 (18%) are within the normative values and 28 (64%) are healthy. The mean values according to AHI distribution are as follows: for mild degree 28.67(±0.58), for moderate degree 27.17 (±2.56) and for severe degree 26.77 (±2.62). These results indicate no statistical significance in their correlation. Among patients with mild-to-moderate OSA, 1 (11%) shows mild cognitive impairments, while the rest of the group are either healthy or within normative values. For those with severe OSA, 6 (20%) have mild impairments. The rest of this group (80%) are healthy or within norm. The mean value for the control group is 28.15 (±1.52), and though this is higher than the OSA group, the correlation between mean values is of no statistical significance.

Even when comparing the controls with the OSA patients who have a severe degree of OSA, the tendency for better cognitive abilities for the control group is still statistically invalid.

The average time for completing part A of the TMT for the OSA group of patients is 43.58 s (±12.84). The scores for part A of the TMT show that 11 (27.5%) of the patients are with severe impairment, 10 (25%) are with mild or moderate, 18 (45%) are within normal and 1 (2.5%) is healthy. In relation to the OSA severity degree, the mean values of the groups are 46.33 s (±18.01) for the patients with mild degree, 40.5 s (±11.89) for those with moderate degree and 43.89 s (±12.79) for those with severe degree. The mean values do not correlate significantly. Among patients with mild or moderate degree OSA there are two patients in each of the groups—severe or mild-to-moderate impairment (a total of 44% of the group) and 5 within normative values (56%). Among those with a severe degree of OSA, 7 (25.9%) show severe impairment, 8 (29.6%) have mild or moderate impairment, 11 (40.7%) are within the norm and 1 (3.7%) is healthy. In comparison to the whole group of OSA patients, the control group has a mean value of 37.54 s (±14.17), which indicates a tendency for better cognitive performance among the controls, which in itself is statistically insignificant (*p* = 0.104). When comparing the controls with the OSA patients with severe degree the tendency does become stronger but remains statistically insignificant (*p* = 0.083).

Upon examining the general cognitive functioning with part B of the TMT, the average time for completing the test for the OSA group is 87.05 s (±32.25). According to scores−13 (32.5%) are healthy, 8 (20%) are within norm, 14 (35%) have mild or moderate impairment and 5 (12.5%) have severe cognitive ability impairment. The AHI scores do not significantly affect the mean values: severe OSA degree−92.59 s (±33.61), moderate OSA degree−63.67 s (±12.29), mild OSA degree−72.67 s (±38.79) (*p* = 0.112). Among the patients with severe AHI scores, 4 (15%) patients have severe impairment, 11 (41%) have mild or moderate impairment, 6 (22%) are within the normal range, and 6 (22%) are healthy. The distribution for mild and moderate degree as per AHI is as follows: 1 patient (11%) with mild or moderate impairment, 2 (22%) within the normal range and 6 (67%) healthy. The mean value for the control group is 76.62 s (±32.61), which is a better overall score than the one for the whole OSA group but does not indicate statistically significant better cognitive functioning (*p* = 0.317). The same correlation is present when comparing the control group to the group with a sever degree as per AHI (*p* = 0.163).

**Figure 2:**
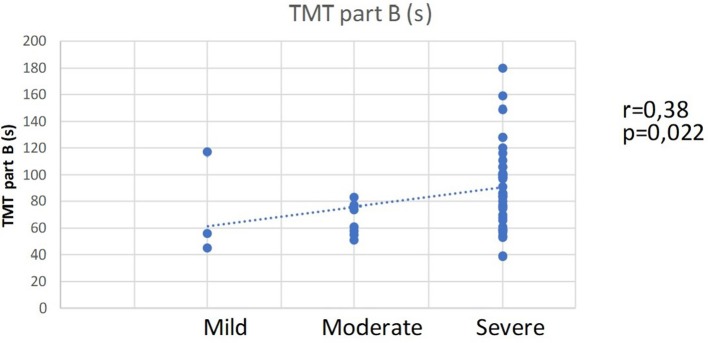
Moderate straight correlation between OSA severity and the scores from TMT, part B.

The correlation analysis for a relation between AHI, BMI and ESS and the scores for the cognitive variables did not indicate a significant correlation. A tendency for a weak to moderate negative correlation between the ESS and MMSE scores (*r* = −0.242, *p* = 0.114) is observed; however, the correlation is not statistically significant. On the other hand, the AHI-values distribution indicates a correlation with the scores from TMT—part B and the distribution according to the degree of impairment by TMT part B. A moderate direct correlation between the AHI-distribution and the TMT, part B scores is established (*r* = 0.38, *p* = 0.022) (Figure 2). This result indicates that the patients with a more severe degree of OSA need more time to complete part B of TMT, which implies the presence of cognitive impairments, as shown by the second correlation.

There is a moderate positive correlation between the distribution of AHI-degree and the distribution of the degree of cognitive impairment according to the scores in TMT part B (*r* = 0.424, *p* = 0.01). This correlation indicates that with increasing severity of OSA, the probability of impairments of attention, psychomotor speed, executive functions and general cognitive functioning increases as well.

#### Statistical properties and analysis of the affective changes among OSA patients

The scores for the OSA group by the Zung Self-rating depression scale give a mean value of 39.66 (±6.236). The results show that in the group of 53 patients, 1 (1.9%) shows severe depressive symptoms, 6 (11.3%) have moderate levels, 15 (28.3%) have mild symptoms, and 31 (58.5%) are healthy.

The mean values according to AHI distribution are as follows: for mild degree, 38.67 (±4.93), for moderate, 38.17 (±8.61), and for severe degree, 40.4 (±5.12). These values are not significantly correlated with one another. According to OSA severity, 1 (11%) mild-to-moderate degree patient shows moderate depressiveness, 3 (33%) patients show mild symptoms, and 5 (56%) are healthy. For the severe OSA degree, 3 (10%) patients indicate moderate depressiveness and 10 (33%) indicate mild depressiveness. The remaining 58.5% of this group are healthy. The mean value from the self-rating scale for the control group is 35.31 (±4.44). The lower results of the control group in comparison to those of the OSA group indicate a statistically significant difference (*p* = 0.011). The tendency for fewer depressive symptoms in the control group increases when comparing the control results with the results of the group with severe OSA (*p* = 0.003).

The mean value for the scores of the OSA group by the NDT-examination is 17.08 (±10.76). According to the scores of 40 participants: 7 (17.5%) are healthy, 16 (36.4%) indicate a pre-neurotic condition, 8 (20%) indicate mild neuroticism, 5 (12.5%)—moderate neuroticism and 4 (10%) indicate severe neuroticism. In relation to the AHI-severity, there is no significant correlation between the mean values: severe OSA– 15.85 (±10.95), moderate−17.33 (±8.52), mild−15.5 (±6.36) (*p* = 0.831). For the group with severe AHI: 3 (11%) patients have severe neuroticism, 2 (7%) have moderate neuroticism, 5 (19%) have mild neurotic state, and 17 (63%) are healthy or in a pre-neurotic condition. The distribution among those with moderate or mild degree AHI is as follows: 1 patient (12.5%) has moderate neuroticism, 3 (37.5%) have mild neuroticism, 4 (50%) are healthy or in a pre-neurotic condition. The mean value of the control group is 9.83 (±4.09), which is a significantly lower score in comparison to the score of the whole OSA group and indicates that depressive and neurotic periods in the control group occur more rarely (*p* = 0.028). The same correlation is observed when comparing the control group with the group with severe AHI, but it is statistically insignificant (*p* = 0.074).

**Figure 3:**
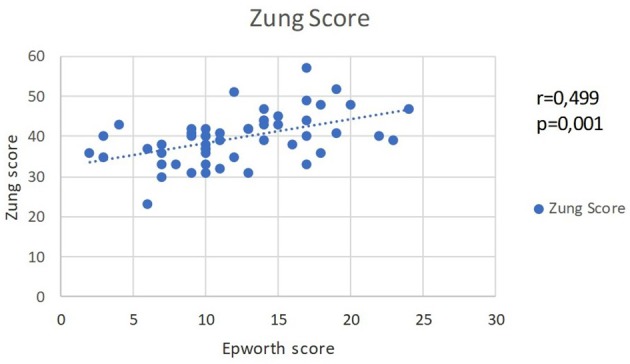
A moderate straight correlation is evident between EES scores and Zung scores.

The analysis of the affective changes, measured by NDT and Zung Self-rating depression scale, do not show a statistically significant relation with OSA and AHI scores. On the other hand, there is a correlation between the two affective scales and the daytime sleepiness scores. Between the scores for Zung Self-rating depression scale and EES scores, there is a moderate-to-strong straight correlation (*r* = 0.499, *p* = 0.001). The association indicates that the experienced daytime sleepiness is associated with depressive symptoms (Figure 3).

**Figure 4:**
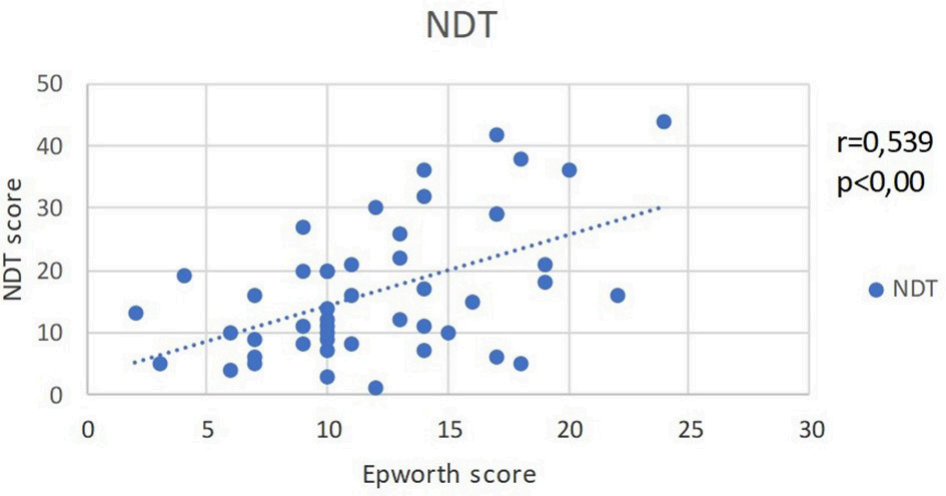
A moderate straight correlation is evident between EES and NDT scores among OSA patients.

EES and NDT scores show an identical strong positive correlation (*r* = 0.539, *p* < 0.001) (Figure 4).

The analysis shows that the increased levels of daytime sleepiness are accompanied by neurotic-depressive symptoms. The relation between AHI and EES scores raises the question about the significance and causes of affective changes in patients with OSA. Additionally, melatonin is significantly involved in the regulation of the circadian sleep-wake rhythm.

## Discussion

OSA is characterized by specific major signs and symptoms that determine the cognitive and affective disturbances experienced by the patients. One of the major symptoms for OSA is excessive daytime sleepiness, which is caused by the disruption of sleep architecture and sleep quality. Daytime sleepiness is considered to be the major and most distinctive symptom of OSA (12). Measuring the severity of daytime sleepiness is a major screening method for diagnosis of OSA. The most commonly used instrument to measure daytime sleepiness is ESS (13). The current study also used ESS to measure the degree of daytime sleepiness among the group of OSA patients in comparison to the control group. According to a vast amount of research data, daytime sleepiness in the general population of healthy individuals has an average value of 4.6 (95% CI 3.9–5.3), with standard deviation of 2.8 (6). The score of our control group is nearly identical to the aforementioned international data: average score−5.15 and standard deviation−2.23. The ESS scores of the OSA group are significantly higher−12.75 (±5.4). Daytime sleepiness, a common symptom of OSA, was found in our patient group. In addition to ESS screening, the patients with OSA underwent a polysomnographic examination for diagnosis confirmation. This instrumental method allowed us to determine the AHI scores, which indicate the severity of OSA. The normative standard for AHI is up to 5. The average AHI score of the group was 58.4 (±30.9). OSA patients were divided into three groups according to AHI scores and the corresponding degree of OSA severity: mild, moderate and severe OSA. There is a noticeable prevalence of the severe OSA patients−81% of all OSA patients. This finding could be attributed to the patients' ignorance or underestimation of their medical condition—a tendency widely discussed in the literature (8, 14–16). Because OSA is hard to diagnose, many clinicians recruit patients for screening who are overweight and obese; these conditions are not directly associated with OSA (12, 13, 15). In the current study, the BMI was computed according to the anthropometrical data of the patients. The BMI values show that all the participants included in the experimental group are overweight, and 95% of them are considered obese. The analysis of the OSA symptoms and severity confirmed the significant correlation between daytime sleepiness and AHI values. The severity distribution shows that severe forms of OSA are more commonly associated with higher levels of daytime sleepiness. The results and norms correspond to the standards provided in the international guidelines (12, 13, 15). On the other hand, the BMI values do not correlate with the AHI values and ESS scores. Overweight and obese status do not necessarily cause sleep-related apnea, as stated in medical literature. The lack of a BMI-OSA correlation is highly revealing because it differentiates the OSA patients from patients with other types of respiratory disorders associated with being overweight, such as Pickwick Syndrome, an alveolar hypoventilation disorder (12, 13, 15). All of this allows us to conclude that the current results are specifically valid for OSA. Many authors associate OSA with cognitive impairment. A wide research data concerning cognitive abilities and functioning among OSA patients is available (5, 13, 17, 18). Bucks et al. using a meta-analysis of 33 studies, conclude that OSA patients demonstrate deficits of attention, memory and executive functions. The authors also take into account the controversial findings about OSA, present in the literature, and come to the conclusion that the verbal abilities and the psychomotor speed remain unaffected by the disorder (5). Our research tools give an account of a broad spectrum of cognitive functioning.

MMSE is a standard screening method for cognitive impairments that has been used to measure the general cognitive functioning of OSA patients. The MMSE scores of the OSA group do show a slight percentage of cognitively impaired patients (11% of the whole OSA group) but do not confirm a statistically significant relation between OSA and cognitive impairment. The relation is also not confirmed by the comparison with the control group. It is possible to attribute the lack of significant relation to the average age of the group. Research by authors such as Gassino et al. and Iglesias et al. report a significant correlation between cognitive impairment measured by MMSE and OSA for older patients (19, 20). The average age of the OSA patients in the current study is 49.43 (±11.79) years. The cognitive scores measured by TMT (Part A and B) are different. The correlation between the scores of TMT, Part B and the AHI severity is significant. The severe forms of OSA are associated with worse test (Part B) performance. The frequency of the cognitive disturbances is also found to be related to OSA severity. The results show that the frequency of cognitive disturbances is higher among patients with severe OSA. These results correspond with the findings of authors such as Aloia et al. Bardwell et al. and Ferini-Strambi et al. who prove the correlation between severe OSA and decreased levels of general cognitive functioning (16, 21–23). We are not able to assess the cognitive functioning of the patients with mild and moderate OSA, because their number is too low. MMSE scores and TMT, Part B scores differ from one another because their compositions are different. TMT, Part B consists of one single task designed to measure a complex of cognitive abilities—the subject should combine multiple cognitive abilities to perform the task. MMSE allows five cognitive domains to be measured by performing 11 tasks. MMSE scores show disturbances in separate domains: orientation, registration, attention and calculation, recall and speech. Part A and Part B of TMT took together, give information about attention, psychomotor speed, executive functions. Part A alone measures sustained attention and psychomotor speed. The current scores show that there are individuals with cognitive disturbances within the OSA group, but these disturbances do not correlate significantly with OSA. A statistically significant difference is also not evident in the comparison of patients' scores and controls' scores. Twigg et al. who do not prove psychomotor speed deficit among OSA patients, presents similar data (24). Quan et al. do not find differences between OSA patients and a control group regarding to their performance on TMT, Part A (25). Both of the cited studies do not take into account the attention properties of OSA patients and are focused only on psychomotor speed. The conclusion made by the authors corresponds to ours and supports the results achieved by Bucks et al. (5).

Another method used in the current research is the 10 Words Memory Test by Luria. It takes into account the subject's attention and its relationship to other cognitive abilities. Ten words to be memorized and recalled is a task designed to measure memory together with the sustained attention and the experienced tiredness during participation. We found that the OSA group demonstrates a significantly lower memorizing ability (attention) in comparison to the control group. This relation implies that patients with OSA are prone to decreased memorizing capacity (fixation). A significant relationship between the disorder itself and the memorizing capacity was not found. This could be attributed to the low number of participants in both groups. We suppose that the enhancement of the sample with more participants will increase the statistical significance of the observed relation. By analyzing the OSA cases separately we found that only seven of them do not demonstrate memory fixation deficits. This tendency corresponds to the one described by Borak et al. and Lee et al. who observe identical correlation among OSA patients in their study (26, 27).

The reproductive memory ability is slightly better among controls in comparison with OSA patients, but the tendency is not statistically significant. Correlation between the disorder itself and the memory reproduction is also not evident. Pierobon et al. come to an identical conclusion in his research devoted to long-term memory (28). Enhancing the sample size would make the tendency clearer. After analyzing the cases separately, we found that more than 50% of the participants do demonstrate deficits of reproductive memory. The tendency observed here means that every second patient with OSA is prone to deficits of attention or long-term memory. The scores concerning the retention memory function are normative for all the patients, while only one of them did not succeed in performing the task 100%. We do not attribute significance to this tendency because the retention function could be reliably measured in a longitudinal study. In the current research, the retention-test as part of Luria's test could be used as additional screening method for OSA patients, where low scores would indicate organic CNS abnormalities, which is an exclusion criteria for the current study. The results of a potential longitudinal study devoted to the retention memory ability could be compared with the conclusions made by Lim et al. who find deficits of retention memory in OSA patients (16). The significant cognitive impairment, registered by TMT, Part B and the significant deficit of the memorizing ability indicate that different attention disturbances are highly expected among OSA patients. After a complex cognitive assessment, Lau et al. also find attention deficits in patients with OSA (29). These results need additional research.

The affective changes in patients with OSA are widely researched. One of the first studies belongs to Guilleminault et al. (3) and show that 24% of male patients with OSA have visited a psychiatrist because of anxious and depressive experiences. In a more recent study, Sharafkhaneh et al. show that 21.8% of a cohort of 118,105 patients with OSA have or have had depressive symptomology (30). Millmann et al. show that 45% of the participants with OSA demonstrate depressive symptoms measured by the Zung scale (31). A previous study by our team confirms the same tendency−40.8% of the included OSA patients demonstrate depressive symptoms measured by the Zung scale (32).

After testing with the Zung self-rating depression scale, 22 patients (41.5%) demonstrate depressive symptoms. Compared with the results of the control group, a difference is apparent, such that there is a higher probability for patients with OSA to develop depressive symptomatic. However, similar to some of our previous research attempts, we could not find a statistically significant correlation between the apnea-hypopnea index and depressive levels, as defined by the Zung-scale. There is a lack of statistically significant correlations between the affective state of the patients and the total duration and the relative share of the different sleep stages. On the other hand, we have found a significant correlation between the results of ESS and depressive symptomatic. The same conclusion is reached by Reynolds et al. (33), who note that there is higher chance of developing a depressive condition for patients with more evident daytime sleepiness. Similarly, in the current research the topic revolves around the examined group of subjects, in which 40% of the patients with OSA do indicate depressive symptomatic. Excessive daytime sleepiness is a leading symptom for diagnosing the disease. The relation between the ESS results and AHI in the current research comes to show that there is indeed a tendency for a correlation between OSA-morbidity and depressive symptomatic. It indicates that there is a higher probability for a patient with a more severe degree of OSA to have depressive symptoms and those symptoms worsen with an increase in the ESS-score. We presume that if we could operate with a bigger sample we would find a direct correlation between the severity of the obstruction and the depressive symptomatic. A further argument in favor of the existence of such a correlation—the results from the NDT-tests shows that 43% of the participants do show depressive-neurotic symptoms. The scores from the NDT in the current research indicate anxiety symptoms in the OSA patients. Borak et al. observe a strong correlation between anxiety and AHI (26). In their research, Sharafkhaneh et al. reach the conclusion that people suffering from an anxiety disorder are more prone to develop OSA (30). Yue et al. perform a study during which they discover that patients with OSA display anxiety more evidently than participants in the control group (34). In their research Sharafkhaneh et al. establish that patients suffering from anxiety disorders are more prone to develop OSA (30). In our research, the OSA patients have also shown higher scores for anxiety and depression compared to the controls. We do not find a direct relation between AHI and anxiety and depressive symptoms, but there is a correlation between AHI and daytime sleepiness. The correlation between NDT and ESS shows that with increasing severity of OSA, the probability of anxiety symptoms is higher and that those symptoms are even more apparent with an increase of the score for daytime sleepiness. Because of the specifics of the research group, we can conclude that there is a correlation between the affective changes in the patients and OSA. Furthermore, with the increase of the OSA severity, the probability of depressive or anxiety symptoms increases as well, whereas the symptoms tend to worsen.

A limiting factor worth mentioning is the small number of participants with mild and moderate OSA. This limitation leads to the conclusion that a bigger and more diverse group of OSA patients is needed for the results of the research to be more definite.

## Conclusion

The current research includes a multidimensional evaluation approach for both objective measurement of physiological functions and subjective assessment of the patients' experiences. We have shown a relationship between obstructive sleep apnea syndrome, presented by certain polysomnographic parameters and the general cognitive functioning and executive abilities impairment. In comparison to the control group without sleep-disordered breathing, the OSA patients demonstrate deficiencies in different domains of the cognition as attention, memory and executive functions. We also observed significantly higher prevalence of depressive symptoms and anxiety among the patients' population compared to the participants in the control group. These symptoms are more frequent than in the general population as well. The main link in this case seems to be the excessive daytime sleepiness experienced by OSA patients. The ESS combined with cognitive impairment could have important social impact especially regarding certain risk groups of the population—professional drivers and people working with machines etc. On the other hand, depressive symptoms can sometimes overlap symptoms of obstructive sleep apnea and thus could remain undiscovered and untreated. This leads to the conclusion that along with the somatic medical condition of the patient, it is of extreme importance that his mental state is treated as well. Due to the many different and contradictory data available on the topic, it is very important that a large database is built in order to aid future prospective, cross-sectional and longitudinal studies of the cognitive abilities and affect of patients suffering from sleep apnea. Alongside this database, the unification of a test battery, which is to be used for diagnosis and treatment control, would further help the recovery of the patients. At this point we can also mention the topic of preventing OSA itself, as well as the need to build a personality profile in order to better assess the morbidity risk at a much earlier stage.

Excessive daytime sleepiness, cognitive impairment and affective disorders are related aspects of social functioning in patients with sleep-disordered breathing and, in their entirety, determine the quality of life. The frequent occurrence of such disorders as part of the complex of symptoms associated with sleep apnea proves their importance, and the evidence of reversibility during treatment, especially in patients with severe disease, necessitates active search, early diagnose, proper treatment and follow up.

The only way to ensure the recovery of patients is teamwork and interdisciplinary approach. Early diagnosis and therapy of respiratory disturbances during sleep are prerequisites for good mental state and social functioning. On the other hand, recognition and treatment of existing breathing disorders during sleep in patients with depressive syndrome or cognitive dysfunction could have a beneficial effect on mental status and play an important role in the therapeutic process. The high prevalence of comorbidity between depressive syndrome, cognitive impairment, and obstructive sleep apnea necessitates new detailed studies of the clinical and therapeutic aspects of the problem and active search for pathogenetic relationships between them. This can significantly improve the diagnosis and therapeutic response.

## Author contributions

DP and OG were responsible for the patient information and informed consent signing. RB and VM interviewed the patients. RB and MN performed the psychological tests. TM, VP, and RB examined the patients and performed the sleep studies. DP, OG, and VM contributed conception and design of the study. RB and MN organized the database and performed the statistical analysis. RB and MN wrote the first draft of the manuscript. DP, VP, and VM wrote sections of the manuscript. All authors contributed to manuscript revision, read and approved the submitted version.

### Conflict of interest statement

The authors declare that the research was conducted in the absence of any commercial or financial relationships that could be construed as a potential conflict of interest. The reviewer VHA and handling editor declared their shared affiliation at time of review.

## References

[1] Management of Obstructive Sleep Apnea/Hypopnea Syndrome in Adults 2003 Edinburgh: British Thoracic Society (2003).

[2] Gastaut H Tassinari CA Duron B. Polygraphic study of the episodic diurnal and nocturnal (hypnic and respiratory) manifestations of the Pickwick syndrome. Brain Res. (1966) 1:167–86. 5923125 10.1016/0006-8993(66)90117-x

[3] Guilleminault C Tilkian A Dement WC. The sleep apnea syndromes. Annu Rev Med. (1976) 27:465–84. 10.1146/annurev.me.27.020176.002341 180875

[4] Sleep-related breathing disorders in adults: recommendations for syndrome definition and measurement techniques in clinical research The Report of an American Academy of Sleep Medicine Task Force. Sleep (1999) 22:667–89. 10450601

[5] Bucks RS Olaithe M Eastwood P. Neurocognitive function in obstructive sleep apnoea: a meta-review. Respirology (2013) 18:61–70. 10.1111/j.1440-1843.2012.02255.x 22913604

[6] Johns MW The Epworth Sleepiness Scale (2013). Available online at: http://epworthsleepinessscale.com/about-the-ess/

[7] Mечков K. Mедицинска психология. Bелико Tърново: ПИК (1995).

[8] Folstein MF Folstein SE Fanjiang G Psychological Assessment Resources Inc Mini-Mental State Examination: Clinical Guide. Lutz, FL: Psychological Assessment Resources (2001). vi, 73.

[9] Mitrushina MN Handbook of Normative Data for Neuropsychological Assessment, 2nd ed, Vol. xxii New York, NY: Oxford University Press (2005).

[10] Кокошкaровa A. Пихологично изследвaне нa личносттa в клиничнaтa прaктикa. София: Медицинa и физкултурa (1984).

[11] Мaджировa Н Пихология и медицинa. Пловдив: МИ Рaйков (2011).

[12] Lurie A. Obstructive sleep apnea in adults: epidemiology, clinical presentation, and treatment options. Adv Cardiol. (2011) 46:1–42. 10.1159/000327660 22005188

[13] Iranzo A Excessive daytime sleepiness in OSA. In: McNicholas WT, Bonsignore MR, editors. European Respiratory Monograph. Plymouth: European Respiratory Society (2011). p. 17–30.

[14] Димитров Б. Психофизиологични изследвaния нa съня и неговите рaзстройствa. София, Бългaрия: Aкaдемично издaтелство “Мaрин Дринов” (2005).

[15] Георгиев О Петровa Д Дихaтелни нaрушения по време нaсън–диaгнозa и лечение: Янковa З., editor. Ново ръководство по белодробни болести и туберкулозa. София: Центрaлнa медицинскa библиотекa, МУ–София (2012). p. 91–100.

[16] Lim W Bardwell WA Loredo JS Kim EJ Ancoli-Israel S Morgan EE. Neuropsychological effects of 2-week continuous positive airway pressure treatment and supplemental oxygen in patients with obstructive sleep apnea: a randomized placebo-controlled study. J Clin Sleep Med. (2007) 3:380–6. 17694727 PMC1978310

[17] Engleman H Joffe D. Neuropsychological function in obstructive sleep apnoea. Sleep Med Rev. (1999) 3:59–78. 15310490 10.1016/s1087-0792(99)90014-x

[18] Findley LJ Barth JT Powers DC Wilhoit SC Boyd DG Suratt PM. Cognitive impairment in patients with obstructive sleep apnea and associated hypoxemia. Chest (1986) 90:686–90. 3769569 10.1378/chest.90.5.686

[19] Gassino G Cicolin A Erovigni F Carossa S Preti G. Obstructive sleep apnea, depression, and oral status in elderly occupants of residential homes. Int J Prosthodont. (2005) 18:316–22. 16052786

[20] Gutierrez Iglesias B Jacas Escarceller C Bardes Robles I Cambrodi Masip R Romero Santo-Tomas O Pujadas Navines F Effectiveness of 6-months continuous positive airway pressure treatment in OSAS-related cognitive deficit in older adults. Behav Neurol. (2013) 26:191–4. 10.3233/BEN-2012-129008 22713425 PMC5215578

[21] Aloia MS Di Dio L Ilniczky N Perlis ML Greenblatt DW Giles DE. Improving compliance with nasal CPAP and vigilance in older adults with OAHS. Sleep Breath (2001) 5:13–21. 10.1007/s11325-001-0013-9 11868136

[22] Bardwell WA Ancoli-Israel S Berry CC Dimsdale JE. Neuropsychological effects of one-week continuous positive airway pressure treatment in patients with obstructive sleep apnea: a placebo-controlled study. Psychosom Med. (2001) 63:579–84. 11485111 10.1097/00006842-200107000-00010

[23] Ferini-Strambi L Baietto C Di Gioia MR Castaldi P Castronovo C Zucconi M. Cognitive dysfunction in patients with obstructive sleep apnea (OSA): partial reversibility after continuous positive airway pressure (CPAP). Brain Res Bull. (2003) 61:87–92. 10.1016/S0361-9230(03)00068-6 12788211

[24] Twigg GL Papaioannou I Jackson M Ghiassi R Shaikh Z Jaye J Obstructive sleep apnea syndrome is associated with deficits in verbal but not visual memory. Am J Respir Crit Care Med. (2010) 182:98–103. 10.1164/rccm.200901-0065OC 20299536 PMC2902762

[25] Quan SF Wright R Baldwin CM Kaemingk KL Goodwin JL Kuo TF. Obstructive sleep apnea-hypopnea and neurocognitive functioning in the Sleep Heart Health Study. Sleep Med. (2006) 7:498–507. 10.1016/j.sleep.2006.02.005 16815753

[26] Borak J Cieslicki JK Koziej M Matuszewski A Zielinski J. Effects of CPAP treatment on psychological status in patients with severe obstructive sleep apnoea. J Sleep Res. (1996) 5:123–7. 8795813 10.1046/j.1365-2869.1996.d01-60.x

[27] Lee J Kim S Lee D Woo J Language function related to sleep quality and sleep apnea in the elderly. Sleep Med. (2009) 10:S50 10.1016/S1389-9457(09)70184-4.

[28] Pierobon A Giardini A Fanfulla F Callegari S Majani G. A multidimensional assessment of obese patients with obstructive sleep apnoea syndrome (OSAS): a study of psychological, neuropsychological and clinical relationships in a disabling multifaceted disease. Sleep Med. (2008) 9:882–9. 10.1016/j.sleep.2007.10.017 18226950

[29] Lau EY Eskes GA Morrison DL Rajda M Spurr KF. Executive function in patients with obstructive sleep apnea treated with continuous positive airway pressure. J Int Neuropsychol Soc. (2010) 16:1077–88. 10.1017/S1355617710000901 20735887

[30] Sharafkhaneh A Giray N Richardson P Young T Hirshkowitz M. Association of psychiatric disorders and sleep apnea in a large cohort. Sleep (2005) 28:1405–11. 16335330 10.1093/sleep/28.11.1405

[31] Millman RP Fogel BS McNamara ME Carlisle CC. Depression as a manifestation of obstructive sleep apnea: reversal with nasal continuous positive airway pressure. J Clin Psychiatry (1989) 50:348–51. 2768203

[32] Bilyukov R Mondeshki T Cherneva R Georgiev O Petrova D Depressive symptoms in patients with respiratory disorders during sleep. Thorac Med. (2011) 3:36–43.

[33] Reynolds CF III Kupfer DJ McEachran AB Taska LS Sewitch DE Coble PA. Depressive psychopathology in male sleep apneics. J Clin Psychiatry (1984) 45:287–90. 6735987

[34] Yue W Hao W Liu P Liu T Ni M Guo Q. A case-control study on psychological symptoms in sleep apneahypopnea syndrome. Can J Psychiatry (2003) 48:318–23. 10.1177/070674370304800507 12866337

